# Motivations for being informal carers of people living with dementia: a systematic review of qualitative literature

**DOI:** 10.1186/s12877-019-1185-0

**Published:** 2019-06-17

**Authors:** Nan Greenwood, Raymond Smith

**Affiliations:** Faculty of Health, Social Care and Education, Kingston University and St George’s, University of London, 6th Floor Hunter Wing, Cranmer Terrace, London, SW17 0RE UK

**Keywords:** Carer, Caregiver, Dementia, Alzheimer’s, Motivation, Qualitative, Systematic review

## Abstract

**Background:**

Informal, often family carers play a vital role in supporting people living with dementia in the community. With ageing populations, the part played by these carers is increasing making it important that we understand what motivates them to take on the role. This systematic review aimed to identify and synthesise qualitative literature describing what motivates people to care for someone with dementia.

**Methods:**

The review followed the Centre for Reviews and Dissemination (CRD) guidelines. Six electronic databases were searched from their first records until August 2018. Synthesis was narrative.

**Results:**

Twenty-six studies fitting the inclusion criteria were identified. Carers described multiple, inter-related motives for caring for someone with dementia. Caring was generally described as a reflection of long-standing family relationships between carers and the care recipients, whether by blood or marriage. Commonly offered motivations included love, reciprocity, filial piety, duty and obligation.

**Conclusions:**

Perhaps the most striking finding was the similarity in these motivations irrespective of gender or relationship with the care recipient. Family relationship and shared history underlay most motivations. Future research should include more longitudinal studies incorporating within study comparisons between different demographic groups to give greater confidence in identifying similarities and differences between demographic groups.

## Background

Across the world, carers play a vital part in caring for their ill and disabled family members and friends. These informal, unpaid, often family carers (or caregivers as they are also known) are commonplace. For example, in the United Kingdom (UK) there are approximately 6.5 million carers – one in eight adults [1]. In the United States of America (USA), the proportion is slightly higher where approximately 18% of the population are carers [2].

Being an unpaid carer is recognised as a mixture of satisfactions [3, 4] and challenges, often with negative effects on carers’ physical and mental health and can result in financial hardship for carers [5]. Carers of people living with dementia are particularly adversely affected by their role and report more mental health problems and stress than other carers [6]. Evidence also shows that the symptoms of dementia in care recipients are strongly associated with carer depression, stress and lower quality of life [7]. Importantly, unlike some caring roles, dementia is a progressive disease with an inevitable decline and although the duration of the caring role varies considerably, it sometimes lasts over a decade [8].

As populations worldwide are ageing, numbers of people living with dementia are rising. For example, it is predicted that the number of people with dementia in the UK will rise from approximately 850,000 to two million by 2050 [9]. Worldwide evidence suggests that most people with dementia live in the community and their primary support is from their families. For example, in the UK, two-thirds of people with dementia live at home, receiving most of their support from family carers [10].

Given the often challenging nature of the caring role, it is important to understand what initially motivates people to become carers of someone with dementia but it is also important to understand why they continue, despite the often growing needs of the care recipient.

Past research has highlighted the significance of carers’ motivations for caring both for carers themselves and those they care for. A review of quantitative literature [11] looked at the impact of carer motivations and ‘meaning’ on the wellbeing of carers of people with dementia. Motivations for caring were described as why carers take on the role, whilst meaning related to how positive the experience of caring was for the carer. Ten studies were identified, six related to meaning and four to motivations. Their synthesis suggested that carers’ kin-relationship to the care recipient and cultural norms influenced motivations to care and that carer wellbeing could be influenced by these motivations. Finding meaning had a positive impact on carer wellbeing. They also reported that carers’ cultural background influenced motivations for caring. Some cultural groups emphasised the role played by religion whilst others highlighted filial responsibilities more than other groups. Overall, the evidence suggested that carers from Western cultures were less likely to refer to filial piety than carers from other cultures. The authors concluded that motivations and meanings can overlap conceptually and that more qualitative investigations are needed in order to understand caring motivations [11].

Importantly, quantitative research has demonstrated that the explanations non-spousal carers offer for caring are associated with care recipient outcomes, relationship quality and carer wellbeing. For example, being unwilling to take on the caring role was associated with higher abusive behaviour towards care recipients [12] and a quantitative questionnaire study found significant relationships between intrinsic motivations, finding meaning in caring and current relationship quality [13]. In another study, family carers of people with dementia who described more external pressure as caring motivators, as opposed to giving personal reasons, also reported more emotional health difficulties [14]. Similarly, Livingstone et al. [15] found carers’ emotional health had an impact on care breakdown, institutionalisation and elder abuse.

Evidence such as this highlights the centrality of carers’ motivations. However, we were unable to identify any recent reviews or syntheses of the qualitative literature. It was therefore decided to focus on qualitative literature to permit synthesis of in-depth research that describes carers’ motivations in their own words and from their perspectives.

## Review aims

1. To identify and describe informal carers’ motivations for caring for people living with dementia, including their motivations at the start of caring and motivations for continuing to care.
2. Where possible to qualitatively identify and describe any similarities or differences in motivations amongst different demographic groups e.g. in terms of gender and relationships (e.g. spouse versus adult child) and ethnic or cultural groups.

## Methods

The review followed the Centre of Reviews and Dissemination (CRD) guidelines [16] and was reported using the Preferred Reporting Items for Systematic Reviews and Meta-Analysis (PRISMA) guidelines [17].

### Electronic search strategy

Six electronic databases were searched without date restrictions: MEDLINE (1946 to 14th August 2018); Embase (1980 to 14th August 2018); PsychINFO (1967 to 14th August 2018); Cumulative Index to Nursing and Allied Health Literature (CINAHL Plus - 1937 to 14th August 2018); Applied Social Sciences Index and Abstracts (ASSIA 1987 to 14th August 2018) and Scopus (1960 to 14th August 2018).

Search strategies were developed according to specific database requirements and consisted of both keywords and Medical Subject Heading (MeSH) terms. Keywords and combinations applied were the same throughout. Table 1 provides the MEDLINE search strategy as an example.

**Table 1:** Search strategy for MEDLINE

Carers	*Caregivers* OR caregiver$ OR care giver$ OR carer$
AND	
Motivations	*Filial piety* OR *Loyalty* OR *Love* OR *Duty* OR *g*iving back OR best placed OR obligat$ OR *Motivation* OR motivation$ OR reason$ OR explanation$ OR rationale OR meaning OR intention to care OR caring intention$ OR caregiving intention$
AND	
Condition	*Dementia* OR dement$ OR *Alzheimer Disease* OR Alzheimer$

MeSH terms are provided in italics

We included both the terms ‘meaning’ and motivations’ in the searches as suggested by Quinn et al. [11]. The terms filial piety and filial obligation are key words in the Quinn et al. paper and so were also included. Other included search terms (e.g. loyalty, love, caring intention and best placed) were found through a combination of thesaurus searching for similar terms and searching of related MeSH terms in Ovid Medline.

### Other sources

Authors of included articles were contacted and asked to identify any additional literature fitting the inclusion criteria. Reference lists of the included studies and relevant systematic reviews identified from the database searches were also examined.

### Inclusion criteria

1. Qualitative studies exploring adult informal, unpaid family carers’ motivations and explanations for providing care for someone with dementia living in the community, for example, in their own homes and not in institutional care.
2. Mixed methods studies where qualitative data could be separated from quantitative data.
3. English language, primary research published in peer-reviewed journals.

### Exclusion criteria

1. Quantitative research and case studies.
2. Studies where less than half the care recipients were diagnosed with dementia.
3. Not peer reviewed, grey literature, reviews, conference abstracts and opinion publications.

### Study screening and selection

Following duplicate removal, both review authors independently screened the titles and abstracts to identify studies potentially fitting the inclusion criteria. Both authors then scrutinised full texts of the selected articles. Where there was uncertainty about inclusion, consensus was achieved by discussion.

### Data extraction and management

Data were entered into standardised tables which included study aims, methods, findings and authors’ conclusions.

### Data synthesis

The research questions were broad, therefore narrative synthesis was selected [18]. Data sources were generally similar with most findings coming from semi-structured interviews thereby reducing some of the potential challenges of multi-method reviews. This synthesis process involved both the review authors independently reading, identifying, recording and summarising the themes relating to caring motivations as described by the original study authors. After this preliminary synthesis, the authors then met and discussed the themes identified and whether, if any, there were identifiable relationships, between the study findings and for example, the participants or geographical locations. In the light of these conversations and the identified motivations, the authors then revisited the included papers again. The very few minor differences between the authors’ analysis after this process were resolved by discussion.

### Quality appraisal

The quality of included studies was assessed independently by both authors using a modified version of a qualitative research rating scale [19]. This scale includes assessment of, for example, whether the methods were appropriate and whether there was clear connection to an existing body of knowledge or wider theoretical framework.

Studies were not excluded based on quality scores but this assessment process allowed interrogation of study methodological quality highlighting strengths and weaknesses.

## Results

Electronic searches identified a total of 3482 articles before duplicate removal: MEDLINE - 838; Embase - 1241; PsychINFO - 702; CINAHL Plus - 168; ASSIA - 282; and Scopus - 251. After duplicate removal, the titles and abstracts of the remaining 2586 articles were scrutinised and 67 full texts were retrieved. From the searches, four relevant systematic reviews [11, 20–22] were retrieved for reference list checking. This process and articles suggested by included study authors revealed 10 additional studies for full text scrutiny.

**Fig. 1:**
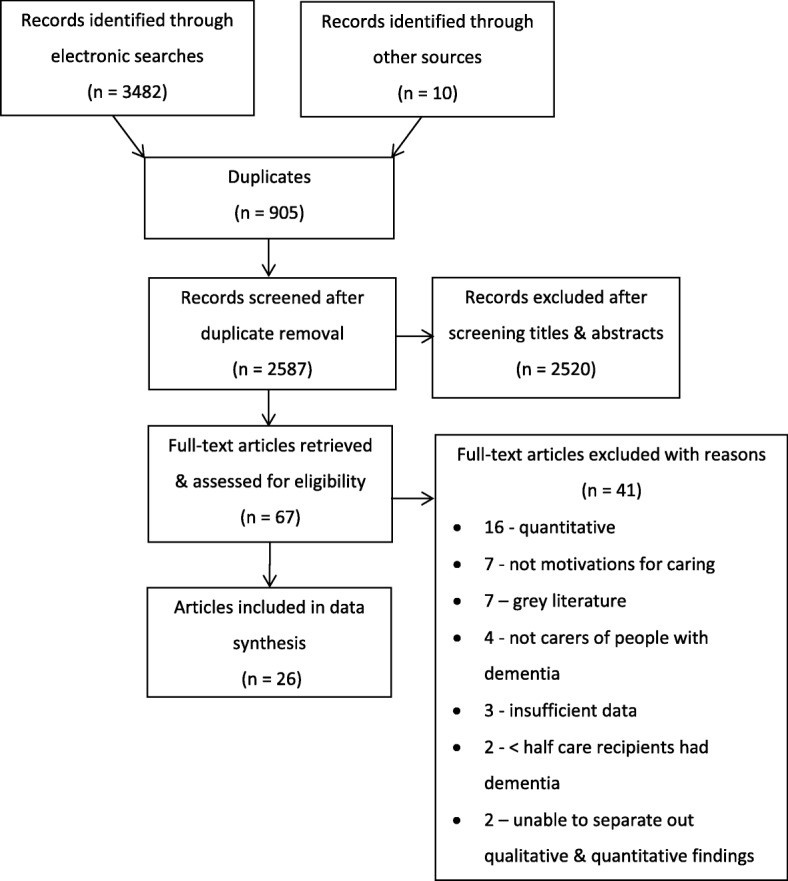
PRISMA [17] flow diagram showing the process of article identification, exclusion and selection

In total 26 studies fitted the inclusion criteria (Fig. 1).

### Studies excluded but which came close to inclusion

Seven studies scrutinised at the full-text stage came close to inclusion but were eventually excluded for the following reasons. Harris [23] and Mukadam et al. [24] provided insufficient relevant data for inclusion. Harris et al. [25] was excluded as less than half the care recipients had dementia. Miyawaki [26] described filial responsibility in carers of frail elderly relatives but the proportion with dementia was unclear. With both Cahill [27] and Kabitsi and Powers [28], it was not possible to separate qualitative from quantitative findings. Caldwell et al. [29] focused primarily on carers’ motivations for care home placements and not for continuing to care.

### Included studies

Twenty-six studies [30–55] published between 1991 and 2017 fitted the inclusion criteria and are summarised in Table 2. Twelve were published since 2010 demonstrating considerable relatively recent research interest in the topic. Most studies came from North America (12) or Europe (8), with the remainder coming from Asia (3), South America (1), Africa (1) and Australia (1). With the exception of one [44] the studies investigated carers of older people with later onset dementia as opposed to younger onset dementia (aged under 65 years). Study aims varied. Most explored carers’ experiences generally, although one [47] specifically explored caring motivations. A total of 761 carer participants (range 5–280) participated in the studies. There were more female [451- (59%)] than male carers [310 (41%)] and participants were mostly spouses and adult children. Eight studies included only spouses and four included only adult children or children-in-law but others included a mixture of relationships. Twelve studies included only co-habiting carers whilst for others the proportion cohabiting was smaller (10–69%) or was not reported.

**Table 2:** Study aims and participant demographics

Authors (date) Country	Aims	Carer numbers	Carer mean age in years (range)	Carer gender (% female)	Carer ethnicity or nationality (%)	Relationship to care recipient (%)	Mean length caring in years (range)	Carer and care recipient co-habiting (%)	Care recipient mean age (range), gender (% male)
Albinsson & Strang (2003) [30] Sweden	To explore issues of freedom, responsibility, existential isolation, death & meaning.	20	59 (42–81)	12 (60%)	NR	Daughter = 9 (45%) Son = 3 (15%) Husband = 3 (15%) Wife = 1 (5%) Brother = 1 (5%) Niece = 1 (5%) Brother in-law = 1 (5%) Daughter in-law = 1 (5%)	NR	2 (10%)	80 years (61–95) 7 (35%)
Cahill (2000) [31] Australia	To develop an understanding of the caring experiences of men looking after spouses diagnosed with dementia.	26	74 (55–87)	0 (0%)	Australian = 26 (100%)	Husband = 26 (100%)	4 years (6–13)	26 (100%)	NR0 (0%)
Chang et al. (2011) [32] USA	To describe factors influencing decisional conflict among Chinese family carers regarding nursing home placement of older adults with dementia.	30	43.9 (25–83)	19 (63%)	Chinese = 30 (100%)	Son = 8 (27%) Daughter in-law = 7 (23%) Daughter = 6 (20%) Spouse = 5 (17%) Grandchild = 2 (7%) Nephew = 1 (3%) Niece = 1 (3%)	3.2 years (1–8)	19 (63%)	NR NR
Eriksson et al. (2013) [33] Sweden	To explore the gender aspects of long-term caring from the perspectives of women providing home care for spouses with dementia.	12	NR (66–80)	12 (100%)	White Swedish = 12 (100%)	Wife = 12 (100%)	NR	12 (100%)	NR 12 (100%)
Gurayah (2015) [34] South Africa	To explore the experiences of those caring for a person with dementia living in rural South Africa.	5	NR (46–68)	4 (80%)	Black African = 5 (100%)	Daughter = 3 (60%) Wife = 1 (20%) Son = 1 (20%)	NR	5 (100%)	NR NR
Harris (1998) [35] USA	To explore the experiences of sons caring for parents.	30	50 (32–71)	0 (0%)	White = 25 (83%) African American = 5 (17%)	Son = 30 (100%)	3.5 years (0.5–11)	17 (57%)	77 years (63–96) 20 (67%)
Harris & Long (1999) [36] USA	To compare Japanese & American men’s experiences of caring & to explore the impact of culture on their role.	45	NR (32–85)	0 (0%)	American = 30 (67%) Japanese = 15 (33%)	Husband (American) = 15 (33%) Son (American) = 15 (33%) Husband (Japanese) = 10 (23%) Son (Japanese) = 5 (11%)	4.5 years (3.5–5)	24 (53%)	American parents: 77 years (NR) Japanese parents: 87 years (71–95) NR NR
Ho et al. (2003) [37] Canada	To explore Chinese-Canadian carers’ feelings about their experiences of caring for relatives with Alzheimer’s disease.	12	54 (30–80)	12 (100%)	Chinese-Canadian = 12 (100%)	Daughter = 8 (66%) Wife = 2 (17%) Daughter-in-law = 2 (17%)	3 years (< 1–7)	7 (58%)	NR NR
Kim (2009) [38] USA	To retrospectively explore the experiences of American-Korean carers of people with dementia & to examine the caring context.	8	67 (48–84)	7 (88%)	Korean-American = 8 (100%)	Daughter/daughter in-law = 4 (50%) Wife = 3 (37%) Husband = 1 (13%)	6 years (2–10)	8 (100%)	81 years (70–95) NR
Lin et al. (2011) [39] UK	To identify, describe & explore changes in carers’ experiences of caring for a relative with dementia & the effects of caring on carer autonomy & health over time.	6	69 (64–72)	3 (50%)	NR	Wife = 3 (50%) Husband = 3 (50%)	NR (6–10) years	6 (100%)	69 years (64–77) 3 (50%)
McDonnell & Ryan (2014) [40] Ireland	To explore the experiences of sons caring for parents with dementia.	13	48 (32–60)	0 (0%)	Caucasian = 13 (100%)	Son = 13 (100%)	NR (2–5) years	13 (100%)	NR (79–95) 1 (8%)
Meyer et al. (2015) [41] USA	To describe the beliefs & experiences of Vietnamese carers of family members with dementia.	10	55 (37–86)	7 (70%)	Vietnamese = 10 (100%)	Spouse = 2 (20%) Adult child = 8 (80%)	NR	All co-habiting (100%)	79 years (69–91) 4 (40%)
Morgan & Laing (1991) [42] Canada	To explore the impact of an Alzheimer’s diagnosis on the spouse 6 months after diagnosis.	9	NR	6 (67%)	NR	Wife = 6 (67%) Husband = 3 (33%)	NR	9 (100%)	NR 6 (67%)
Murray et al. (1999) [43] Europe – multiple countries	To explore the aspects of dementia which spouse carers find most difficult, rewards of caring & national & gender differences.	280	71 (55–79)	162 (58%)	NR	Wife = 162 (58%) Husband = 118 (42%)	NR	280 (100%)	73 years (61–81) 162 (58%)
Pang & Lee (2017) [44] Hong Kong SAR, China	To explore the caring experience of spousal carers of people with young onset dementia in Hong Kong.	6	67 (61–73)	3 (50%)	Chinese = 6 (100%)	Wife = 3 (50%) Husband = 3 (50%)	3.5 years (1–6)	6 (100%)	58 years (52–63) 3 (50%)
Peacock et al. (2010) [45] Canada	To describe the positive aspects of caring identified by carers of people with dementia.	39	NR	32 (82%)	NR	Spouse = 22 (56%) Adult child = 13 (33%) Other = 4 (11%)	NR	NR	NR 25 (68%)
Qadir et al. (2013) [46] Pakistan	To explore dementia awareness among carers, their attitudes toward family members with dementia & experiences of burden.	12	34 (19–47)	7 (58%)	Pakistani = 12 (100%)	Daughter = 5 (42%) Son = 3 (25%) Daughter in-law = 2 (17%) Grandson = 1 (8%) Nephew = 1 (8%)	NR	NR	73 years (55–90) 8 (66%)
Quinn et al. (2015) [47] UK	To explore how meaning, motivation & relationship dynamics combine to influence experiences of dementia caring.	12	65 (41–86)	10 (83%)	White British = 12 (100%)	Spouse = 8 (67%) Daughter = 4 (33%)	NR	12 (100%)	76 years (41–88) NR
Russell (2001) [48] USA	To explore what male carers do, the meanings they ascribe to their work & their strengths & vulnerabilities.	14	NR (68–90)	0 (0%)	White American = 11 (79%) White European = 2 (14%) African American = 1 (7%)	Husband = 14 (100%)	NR	NR	NR 0 (0%)
Santos et al. (2013) [49] Brazil	To explore differences in disease awareness in Latin American carers of people with dementia.	18	61 (NR)	16 (89%)	Brazilian (100%)	Daughter = 10 (56%) Spouse = 6 (33%) Distant relative = 2 (11%)	NR	18 (100%)	78 years (NR) 7 (39%)
Siriopoulos et al. (1999) [50] Canada	To investigate the experiences & needs of husbands caring for wives with Alzheimer’s disease.	8	NR (64–92)	0 (0%)	White (100%)	Husband = 8 (100%)	NR (< 1–10) years	6 (75%)	NR (68–90) 0 (100%)
Sterritt & Pokorny (1998) [51] USA	To explore the meaning of caring & describe African-American carers’ experiences of caring for family members with Alzheimer’s disease to see how cultural attitudes, beliefs & values affect experiences.	9	54 (31–80)	8 (88%)	African-American (100%)	Daughter = 5 (56%) Brother = 2 (22%) Wife = 1 (11%) Granddaughter = 1 (11%)	4.8 years (3–8)	NR	77 years (61–88) NR
van Wezel et al. (2016) [52] Netherlands	To explore perspectives of female Turkish, Moroccan & Surinamese Creole carers in the Netherlands of relatives with dementia & to explore similarities between these groups.	69	NR (20–84)	69 (100%)	Turkish = 26 (38%) Moroccan = 26 (38%) Surinamese = 17 (24%)	Daughter = 55 (80%) Daughter in-law = 9 (13%) Wife = 3 (4%) Other = 2 (3%)	NR	21 (30%)	NR NR
Vellone et al. (2002) [53] Italy	To improve understanding of the experiences of Italian carers of people with Alzheimer’s disease.	26	57 (35–86)	20 (77%)	Italian = 26 (100%)	Spouse = 19 (73%) Adult child = 7 (27%)	5 years (2–9)	NR	NR NR
Wallhagen & Yamamoto-Mitani (2006) [54] USA	To compare & contrast cultural influences & familial role expectations of Japanese carers of older adults with dementia in Japan with American carers of older adults with dementia in the USA.	16	Japanese = 54 (47–57) American = 49 (41–63)	16 (100%)	American = 9 (53%) Japanese = 7 (47%)	Daughter = 12 (75%) Daughter in-law = 3 (19%) Niece = 1 (6%)	NR	NR	Japanese care recipient = 82 years (NR) NR American care recipient = 80 years (NR) NR
Yamamoto & Wallhagen (1997) [55] Japan	To develop concepts to facilitate understanding of why carers of people with dementia can continue providing care despite difficulties.	26	NR (32–63)	26 (100%)	Japanese = 26 (100%)	Daughter = 13 (50%) Daughter in-law = 13 (50%)	NR	18 (69%)	NR (63–99) NR

*NR* Not reported

With the exception of six studies [30, 31, 39, 42, 43, 45], carer ethnicity, cultural group or nationality were reported. Fifteen studies focussed on only one ethnic or cultural group, six of which included only Far Eastern Asian carers [32, 37, 38, 41, 44, 55]. Nine other studies also included only one cultural group. These were sometimes reported by nationality, e.g. Australian [31] or White Swedish [33]. Other groups included: Black African [34], Caucasian [40], Pakistani [46], White British [47], Brazilian [49], White Canadian [50], African American [51] and Italian [53]. Five studies included more than one ethnic or national group [35, 36, 48, 53, 54]. Care recipients were less well described than carer participants and, unsurprisingly given the carers’ relationships described above, they were frequently spouses or parents. Full carer participant demographic details are available in Table 2.

Most studies used purposive sampling with face-to-face, semi-structured and in-depth interviews (Table 3). The overwhelming majority were cross-sectional although there were four longitudinal studies [33, 39, 42, 54]. Analysis varied but was commonly thematic or content analysis. Study quality (maximum 12) was generally good (median 9) but was variable. Ratings ranged from four [51] to 11 [30, 32, 35, 37, 40, 44, 47, 55]. Most studies scored between 7 and 10. Lower scores were usually due to limited or missing details about methodological design, analysis and researcher reflexivity (Table 3).

**Table 3:** Study methods and quality scores

Authors (date)	Sampling	Data collection (all face-to-face and one-to-one unless otherwise specified)	Theoretical background Data analysis	Quality scores (max 12)
Albinsson & Strang (2003) [30]	NR	Cross-sectional, interviews	NR Data categorisation based on hermeneutic approach	11
Cahill (2000) [31]	Non-probability	Cross-sectional, semi-structured interviews	NR NR	7
Chang et al. (2011) [32]	Purposive	Cross-sectional, semi-structured interviews	NR Thematic analysis	11
Eriksson et al. (2013) [33]	NR	Longitudinal, interviews	Feminist perspective ‘Analytic framework’	7
Gurayah (2015) [34]	Purposive	Cross-sectional, semi-structured interviews	NR Thematic analysis	6
Harris (1998) [35]	Purposive	Cross-sectional, in-depth interviews	NR Content analysis	11
Harris & Long (1999) [36]	Purposive	Cross-sectional, in-depth interviews	NR NR	6
Ho et al. (2003) [37]	Purposive	Cross-sectional, in-depth, semi-structured interviews	Stress model Thematic analysis	11
Kim (2009) [38]	Purposive	Cross-sectional, in-depth, semi-structured interviews	Transcendental phenomenology Transcendental phenomenological analysis	10
Lin et al. (2011) [39]	NR	Longitudinal, semi-structured interviews & observations	Grounded theory Constant comparative analysis	6
McDonnell & Ryan (2014) [40]	Purposive	Cross-sectional, in-depth, semi-structured interviews	Colaizzi’s (1978) ‘seven-stage process’ to identify themes	11
Meyer et al. (2015) [41]	Snowball	Cross-sectional, in-depth, semi-structured interviews & 1 focus group	NR Thematic analysis	9
Morgan & Laing (1991) [42]	NR	Longitudinal, unstructured interviews	Grounded theory Constant comparative method	7
Murray et al. (1999) [43]	NR	Cross-sectional, semi-structured interviews	Grounded theory Content analysis	7
Pang & Lee (2017) [44]	Purposive	Cross-sectional, in-depth interviews	NR Content analysis	11
Peacock et al. (2010) [45]	Purposive	Cross-sectional, 6 focus groups & 3 interviews	NR Thematic analysis	9
Qadir et al. (2013) [46]	NR	Cross-sectional, in-depth, semi-structured interviews	NR Thematic analysis	7
Quinn et al. (2015) [47]	NR	Cross-sectional, semi-structured interviews	Interpretative phenomenological analysis (IPA) Thematic analysis	11
Russell (2001) [48]	Purposive	Cross-sectional, in-depth, open-ended interviews	NR Inductive analysis	7
Santos et al. (2013) [49]	Convenience	Cross-sectional. Analysis of session transcripts (transcribed from)	Interpretative phenomenological analysis (IPA) Qualitative analysis	7
Siriopoulos et al. (1999) [50]	NR	Cross-sectional, semi-structured interviews	NR Giorgi’s (1985) phenomenology method	7
Sterritt & Pokorny (1998) [51]	Purposive	Cross-sectional, open-ended question interviews	NR Thematic analysis	4
van Wezel et al. (2016) [52]	NR	Cross-sectional, individual interviews & focus groups	NR ‘Generic qualitative approach’ to identify themes	9
Vellone et al. (2002) [53]	NR	Cross-sectional, interviews	Phenomenology Phenomenological analysis to identify themes	9
Wallhagen & Yamamoto-Mitani (2006) [54]	NR	Longitudinal, semi-structured interviews	NR Constant comparative techniques to identify themes	9
Yamamoto & Wallhagen (1997) [55]	Theoretical	Cross-sectional, interviews	NR Constant comparative approach to develop categories	11

*NR* Not reported

To help answer the review questions, the synthesised findings are firstly presented overall and then by carer demographic characteristics (relationship, gender, ethnicity, nationality, culture and religion). Where studies included a variety of relationships with the care recipients, motivations were often described for participants together and it was not always possible to be confident whether the motivations related to specific relationship groups. Therefore, only where it was clear to whom the motivations applied, are the motivations described specifically as relating to one relationship group. It also proved difficult to separate out descriptions of motivations to care in general as distinct from motivations for continuing to care. However, where this was possible, findings describing why carers continue in their role are summarised in the final section.

Full details of the studies’ main findings are provided in Table 4.

**Table 4:** Study findings

Authors (date)	Motivations for caring overall	Motivations identified for continuing to care	Authors’ conclusions
Albinsson & Strang (2003) [30]	Motivations included obligation & feelings of guilt, being faithful, reciprocity, responsibility & having always taken care of others.		This study underlines the importance of not only identifying carer physical & psychosocial features but also existential ones. Staff need to be more aware of these issues in order to support families also in existential crisis.
Cahill (2000) [31]	Husbands’ motivations included love, marriage & duty or a combination of all three. They also reported reciprocity & commitment to their marriage vows & religious obligation was also important.		Men reported wanting to ‘do the right thing’ & continue caring for their wives at home. Men & women are similar in their motivations to care.
Chang et al. (2011) [32]	Placing relatives in a care home was seen as a violation of filial piety. Some worried that doing so could be seen as ‘un-filial’ by family members. Placement decisions were influenced by filial piety, limited financial resources & information, older adults’ preferences, family disagreement, distrust of nursing home care quality & limited availability.	Carers chose to continue caring due to distrust of nursing home quality & the perception that nursing homes are where people go to die.	Nursing home placement continues to contradict the Chinese value of filial piety causing decisional conflict for many carers. To understand this process among Chinese carers, filial piety & collectivism, traditional & changing family carer roles & nursing home quality & care quality all need to be considered.
Eriksson et al. (2013) [33]	Motivations & commitment to caring were based on the carers female identity – they had always cared for their families & this is an extension of this.		Caring experiences relate to society’s expectations about women’s roles. Women view their caring role & responsibilities as paramount. Other duties, including caring for themselves, are deemed less important. The intense commitment & responsibilities experienced by female carers must be acknowledged.
Gurayah (2015) [34]	Duty to provide care for family members was seen as part of African culture, with both a sense of obligation & responsibility.		Caring for parents & family is implicit in African culture & is embodied in the concept of Ubuntu. Individuals are part of the community & need to fulfil their obligations to the collective. Caring is seen as character-building.
Harris (1998) [35]	Sons reported a sense of duty, responsibility, love & filial obligation. Being a dutiful son was a driving force.		Sons were motivated by a sense of love & or obligation. This did not depend on whether there was a sister to provide care.
Harris & Long (1999) [36]	Sons: Japanese sons reported an obligation to care for their parents based on filial piety. It was most often considered the eldest son’s responsibility to care for a parent. For American sons, birth order played little part, instead they reported caring out of love, commitment & duty. Husbands: Japanese husbands reported caring out of commitment, love & reciprocity. It was thought natural for them to take on the caring role. American husbands also reported caring out of reciprocity & love but in addition talked about upholding marriage vows.	The same traditional values were also motivators for providing care as opposed to placing relatives in nursing homes.	Culture shapes caring experiences. Japanese sons spoke frequently about filial responsibility. The Japanese multigenerational structure influences obligation to provide care, with children taking on caring responsibilities & parents assuming their children will care for them. American husbands talked about wedding vows & believed their children have their own lives & do not expect them to provide care.
Ho et al. (2003) [37]	Traditional Chinese cultural values instilled an obligation & responsibility to provide care for family members. Due to filial obligation, many carers expected to be carers at some point. Participants compared themselves positively to Western attitudes to care.		Providing care is part of Chinese culture. Many seemed distressed by the apparent inconsistency between traditional ways & the reality of caring. Although they accessed outside support & were considering care home placements, this was discussed as part of becoming assimilated into Western culture.
Kim (2009) [38]	Motivations included the continuation of marital relationship, love, making the cared for comfortable & filial piety derived from cultural norms. Caring was seen as a ‘family affair’.	Caring was described as a continuation of marriage. Love helps them to continue caring.	Caring is determined by multiple motives: filial piety, the availability of carers, the history of communal relationships & attachment to elderly parents. Obligation seemed insufficient motivation for caring for an older person with dementia.
Lin et al. (2011) [39]	Spouses described caring out of emotional commitment, happiness derived from caring, love, responsibility & duty (moral & societal obligations) & reciprocity. Four categories emerged: my life changed, commitment, responsibility, duty & support. My life changed represented the beginning of the caring journey. Learning from experience offered new perspectives on carers’ experiences. Responsibility and duty increased over time but the support from formal & informal sources fluctuated. All carers experienced changes in the caring journey.		Moral & societal obligations are linked to a sense of duty, with deciding to provide care influenced more by societal expectations than innate desire to care.
McDonnell & Ryan (2014) [40]	Sons cared for their parents out of a sense of love, devotion, loyalty & respect. They reported a strong sense of duty & satisfaction. Reciprocity was also highlighted.		Devotion & willingness to care for parents were highlighted. However, the study took place in rural Ireland & farm succession plans may have played a role.
Meyer et al. (2015) [41]	Underlying all themes was the idea that cultural beliefs, values & expectations impacted on caring experiences. Caring was motivated by filial piety. Placing the care recipient in a care home went against Vietnamese culture. Some cared out of love & affection & some out of guilt. Carers highlighted reciprocity for sacrifices their parents made, especially related to the Vietnam war.		An overarching theme was that cultural beliefs, values & expectations impacted on caring experience. Differing levels of acculturation sometimes led to family conflict as younger family members did not always see it as their duty.
Morgan & Laing (1991) [42]	Carers fitted into either ‘grief’ & ‘role strain’ groups. In the ‘grief’ group, spouses cared out of love & wanting to provide a sense of normalcy & continuity. Many of them also suggested that reciprocity was a motivation. In the ‘role strain’ group responsibility & duty were the main motivations.		Health care professionals need to be aware of how past relationships can influence their attitude towards caring.
Murray et al. (1999) [43]	Motivations included reciprocity for past care (12%), the desire for continued companionship (16%), ‘job satisfaction’ (16%) & the fulfilment of duty (39%).	Continued companionship & the satisfaction of providing care were motivations for continuing to care.	Spousal carers in the 14 countries described the difficulties & rewards of caring in similar terms. This suggests commonality of experience, in spite of diversity in informal & statutory provision of care for older people between different countries.
Pang & Lee (2017) [44]	Spouses believed caring was part of their marital obligation. Spiritual & religious beliefs were also described, with some describing caring as their destiny & that everything in life was already planned out for them. Their religion also motivated them to continue their caring role. Both husbands & wives described repaying the care or support they had received from their spouses in the past as a motivation for caring.	Providing good quality care led to a sense of satisfaction for the carers which motivated them to continue caring.	In Chinese culture, couples are bound to a martial philosophy which asserts a lifelong commitment to mutual care. Carers were resigned to their caring role as they believed everything in life is pre-determined. Carers focused more on the positives & meaningful side of caring, not on losses. They felt satisfied & were more motivated to continue caring.
Peacock et al. (2010) [45]	All carers described positive aspects of caring. In particular, it provided them with the opportunity to for reciprocity to the family member out of love & wanting to make them as comfortable as possible. Spouses talked about caring being part of marriage, however husbands discussed repaying wives for past care whilst wives saw it as a continuation of the relationship. Many reported doing all they can so the person with dementia can stay at home as long as possible.		Caring for a family member is full of opportunities, e.g., being able to ‘give back’, to discover personal strengths & become closer to the person with dementia. Spouses viewed caring as an opportunity to continue in their marital relationship despite numerous challenges.
Qadir et al. (2013) [46]	Caring was viewed as an obligation & part of their duty. It was influenced by their religious (Islam) obligation to care for relatives & ‘worthy of divine reward’. Reciprocity was also important & was expected to be rewarded by prosperity & success.		In Islamic society, displaying respect towards parents is part of worship of God & is an obligation highlighted in the Quran. Reverence towards parents & closeness to family are emphasised in early childhood & continues to be important throughout life.
Quinn et al. (2015) [47]	Carers’ relationship with their relative was the primary reason for taking on caring. Reciprocity & appreciation of their help gave caring meaning. Caring was seen as *‘something you just get on with’* & a natural continuation of relationships. Some also felt they had no choice, that it was their ‘job’, & no one else could do it. They would feel guilty if they were to place the cared for in an institution.	Carers often had no choice but to continue providing care as no one else could do it & they saw it as their ‘job’.	Carers need to find meaning from providing care & derived this from believing it was their responsibility to provide care & they were reciprocating past help from relatives. The relationship with the relative was the primary motivation for taking on the caring role.
Russell (2001) [48]	Husbands reported caring out of commitment, devotion & responsibility. Many husbands felt their commitment to providing care prevented their wives from needing care home placements. Some talked about reciprocity for the previous care their wives had given them.		While men may experience isolation & feelings of being an ‘invisible’ carer, they exhibit powerful feelings of commitment as well as adaptability & resilience.
Santos et al. (2013) [49]	Motivations included duty, responsibility, feelings of gratitude, reciprocity, familism (family caring is regarded as natural even if the pre-morbid relationship was not good) & religiosity.		Motivations to provide care are determined by both cultural & individual aspects & may influence disease awareness. Family caring may be motivated by affection, altruism, social norms of responsibility & also egotistic motivation.
Siriopoulos et al. (1999) [50]	Motivations included feeling obliged to reciprocate care provided by their wives, love & belief in their marriage vows. Husbands were devoted to caring for their wives.		The quality of the relationship was important for husbands in deciding to care. Husbands felt a sense of reciprocity due to the love & dedication of their wives throughout their marriage. Spouses’ demographic characteristics appeared to have no effect on their attitudes to caring.
Sterritt & Pokorny (1998) [51]	Caring was seen as a ‘traditional family value’, was the right thing to do & was something they could feel good about. They also cared out of love & found it rewarding. Caring was seen as a female role & men were not expected to provide care.		More cultural awareness of reasons for providing care needs to be incorporated into training nurses & physicians. These efforts may strengthen & enhance the existing use of support networks & provide culturally congruent care.
van Wezel et al. (2016) [52]	Caring for a family member was described as superior to professional care & was imposed by religion & culture. Reciprocity was important but many younger carers also viewed it as an obligation. Others said caring was a test from God or Allah or that providing care was seen as a duty & a role for women.	Carers continue caring because they view family care as superior to professional care, e.g. more loving & better meets the care recipients’ cultural & religious needs.	Carers see caring as something that should be performed with respect & love. Caring is the act of a ‘good religious person’. They derive great satisfaction from caring. This fulfilment largely outweighs the burden of care.
Vellone et al. (2002) [53]	Family duty was described as an important.		Italians see it as their duty to care. Caring for people with Alzheimer’s disease at home is consistent with their culture.
Wallhagen & Yamamoto-Mitani (2006) [54]	There were many commonalities between the Japanese & American carers. Both referred to reciprocity, moral obligation & responsibility. The word obligation was not used by Japanese carers who described it as a ‘matter of course’. More Japanese carers discussed moral obligation to care & their culture. Japanese carers said their family position meant they had to adopt the role - it was an expected ‘career’. This was not so for American carers.		The finding that reciprocity & attachment as motivations to provide care are supported by previous research. Japanese culture recognises & praises caring activities, with Japanese carers deriving a strong sense of fulfilment, pride & self-worth. American carers, by contrast viewed caring as an unexpected career & without the same level of societal praise.
Yamamoto & Wallhagen (1997) [55]	Three societal norms influence filial piety in Japan: filial responsibility, beliefs regarding women’s role which attributes high value to caring & family position (daughters in-law of first born sons expect to take on this role). Caring was motivated by reciprocity for past parental care & was also felt by daughters in-law on behalf of her husband. Emotional bonds between the carer & care recipient, the carer’s sense of achievement & gratitude from care recipients were also important.	Daughters continue to care in order to live free of regret when caring ends, remain committed to decisions made & because of religious beliefs. Rewards are also financial (e.g. avoiding nursing home costs).	Societal norms have a strong conforming power in Japanese society & the norm of filial responsibility influenced daughter & daughter in-law carers. Rather than saying they become carers because there is nobody else to take on the role, most Japanese carers felt they were expected to provide care, due to the norms in Japan which honour caring as part of women’s roles.

#### Overall

Carers described multiple, inter-related motives for caring for someone with dementia. Motivations did not appear to have any association with study publication date or study quality. Caring was generally described as a reflection of long-standing family relationships between carers and care recipients whether by blood or marriage. Commonly offered motivations included love, reciprocity, filial piety, duty and obligation. Some carers emphasised caring through choice whilst others described feeling obliged to take on the role whether this was from societal, cultural or family pressures or because available formal care was perceived to be poor quality or expensive. Some carers also said they were in the caring role to avoid care home admission [32, 36, 41, 45, 48, 55]. Avoidance of guilt was also a motivator [30, 41, 47].

#### Relationships

Overall spousal and adult children carers described very similar caring motivations. Spousal carers often reported caring out of a mixture of reciprocity, commitment to marriage, love, duty and responsibility [31, 36, 38, 39, 42, 44, 50, 53]. Similarly, adult children and children in-law mentioned reciprocity, family values, love, filial piety, loyalty, duty, obligation and responsibility [34, 36, 37, 40, 41, 46, 49, 51, 52, 54, 55]. Providing care within the family was also seen as superior to professional care [52]. The rewards of caring were highlighted in some studies of spouses [39, 43], sons [40] and daughters [55].

#### Ethnicity, nationality, culture and religion

Although there were some differences, motivations for caring were largely similar across ethnic and cultural groups. Many studies did not specify the ethnic or cultural groups of their carer participants, therefore in the following section, where ethnic or cultural groups were not supplied, review findings are reported by study country.

Reciprocity, love, duty, marital commitment and responsibility were frequently highlighted across carers in all included studies. However, the satisfactions derived from caring were more often highlighted by carers from Western countries [39, 40, 43] but it was also mentioned by Pang and Lee [44]. The term filial piety was more frequently used by authors describing the motivations of carers from Far Eastern groups such as Japanese [36, 55], Chinese [32], Korean [38] and Vietnamese [43]. However, other authors referred to e.g. filial obligation [35, 37]. Many explicitly emphasised the importance of family members caring for each other in their cultures as a reason for caring [33, 34, 37, 38, 49, 51, 53].

With the exception of three studies [36, 52, 54], few made clear within study comparisons between ethnic and cultural groups. Those that did suggest that, although there are some differences between groups, generally there are more similarities than differences.

Avoidance of nursing home admission was mentioned in studies that included carers from several ethnic and cultural groups including Chinese [32], Vietnamese [41], British [47] and Dutch [52].

Qadir et al. [46] reported that duty and obligation were influenced by the carers’ Islamic faith. However, although religion and caring motivations were highlighted in several studies from several countries, the precise religion was not always specified [31, 44, 49, 52, 55].

#### Gender

Many of the motivations to care were similar for men and women and included reciprocity, love, responsibility and duty. However, caring was sometimes explicitly linked to being wives, daughters or daughters-in-law with some females describing caring as part of general female caring role within the family [51, 54] or part of their female identity [33]. For Japanese women, being a carer was a *‘matter of course’* (p69, [54]). Three studies offered insight into son carers [35, 36, 40]. For Japanese sons, birth order was important with the eldest typically responsible for providing care for relatives [36]. Findings from the other two studies suggested that sons, like other carers, were largely motivated by love and duty [35, 40].

### Continuing to care

It was not always possible to separate reasons for continuing to care from more general motivations but where it was possible, findings suggested a range of motivations for continuing to care. Some of these were the same as more general reasons for caring, for example, love and duty and part of their marital relationship [36, 38]. Other reasons included avoiding paying for nursing homes [55], distrust of nursing home care or regarding their care as superior to institutional care [32, 52]. The belief that carers could provide better care than professionals as they would care with more love and would provide care more tailored to individual needs [52] was also highlighted. Continuing companionship and satisfaction [43, 44] and the belief that no one else was available [47] or that they would regret it if they did not continue in the role [55] were also highlighted.

## Discussion

This review brought together studies from a wide range of cultures and countries covering nearly three decades of research. Many recent studies published since 2010 were identified suggesting a growth in interest in the topic. There were usually multiple explanations for caring but perhaps the most striking finding was the apparent similarity in carers’ motivations for caring irrespective of their relationship with the care recipient, country of origin, ethnic or cultural background or gender. Motivations highlighted were frequently based on long-standing family relationships and emotions such as love and wanting to reciprocate or return care received from the person with dementia in the past. Caring was not generally regarded as something extraordinary but rather as a natural part of family life [36] and something *‘you just get on with’* (p229, [47]). Importantly, carers had multiple motivations for caring and it was rarely driven by one motivation alone. This is perhaps not surprising. Caring for someone with dementia is a fluid, complex, often long-term role and it is likely that the motivations and the mixture or balance of motivations change as the health of the person with dementia, the carers’ own health and their situations change.

There were only four longitudinal studies [33, 39, 42, 54] and these tended not to emphasise any changes over time. However, Lin et al. [39] described how carers learnt from their caring experiences whilst responsibility and duty increased over time but support from formal and informal sources fluctuated.

Our review and the one by Quinn et al. [11] have shown that cultural factors play a role in motivations for being a carer but importantly emphasis on cultural differences may be misleading. Throughout these studies caring was underlain by long-standing relationships, often between close family members irrespective of carers’ cultural background. This is a significant finding as it suggests motivations for caring may not be so very different across cultures as much of the literature appears to suggest. Caring was usually underwritten by love and respect for the person with dementia. Although some investigations did make specific within study ethnic or cultural comparisons, these were in the minority and they reported more similarities than differences in motivations [36, 52, 54].

Caring was frequently described as duty, obligation or responsibility. Filial piety or filial obligation was often explicitly given as an explanation for caring in studies including carers originally from China [32, 37] Korea [38], Vietnam [41] and Japan [54, 55]. Filial piety can be defined as a principle in Chinese and other Asian cultures, which *‘emphasizes honour and devotion to one’s parents… implies that adult children have a responsibility to sacrifice individual physical, financial and social interests for the benefit of their parents or family*’ (p14, [22]). However, carers from a wide range of other countries including South Africa [34], the USA [35], the UK [39], Pakistan [46] Brazil [49] and the Netherlands [52] also referred to caring for very similar reasons to those relating to filial piety, such as duty, obligation and responsibility. Again, this emphasises the commonalities across cultures.

### Limitations of the included studies

The quality of the studies was variable with many omitting to report important methodological and participant demographic details. However, we did not detect any associations between study quality and reported findings. Synthesis of the studies was hampered by the fact that some studies did not report details such as carer ethnicity and when they did, they did not always present them in the same way. This meant that we often had to rely on the country of data collection and we were sometimes unable to comment on the relationship between ethnicity and motivations. Future research should ensure such details are reported.

Study findings were not presented in a manner that permitted identification of what, if any, motivations were seen as the primary or most important motivations by carers and although, perhaps tempting, it is important not to assume that the most frequently mentioned motivations were the most significant and carried the most weight for carers. The carers here may have responded with motivations that they perceived were most socially acceptable and the motivations offered may not have been the most important reasons for caring for them.

Indeed, carers may also not often consciously consider what motivates them to care for others, perhaps just regarding it as an unquestioned part of family relationships [19]. This may make it harder for them to articulate their motivations in research interviews and as a result, they may have relied on socially desirable explanations such as ‘love’ or ‘duty’. Furthermore, social desirability may have made it difficult to admit to not wanting to care or choosing to care to save money on care home costs.

Any study investigating carers’ experiences is hampered by the fact that many people that researchers and service providers regard as carers may not see themselves as carers. For them, caring may be seen as a progression in their relationship [56] and they may make few, if any, clear distinctions between spousal or kinship support as opposed to carer support [19]. They therefore may not volunteer to participate in research and as a result their experiences may not be reported. This important group of unidentified carers requires greater investigation in order to capture the motivations of all carers.

### Review strengths and limitations

Our review was comprehensive and wide reaching and included many diverse studies. We believe the decision to include both ‘motivation’ and ‘meaning’ as search terms was justified given the conceptual overlap in the two concepts [11]. The decision to exclude quantitative studies to allow carer participants to describe their motivations in their own words, excluded potentially relevant studies. Despite this, 26 studies were included in the review.

There were some challenges to undertaking the review. For instance, although most studies only collected data using single one-to-one interviews, variability in reporting the data made synthesis more challenging. We also attempted to further understanding of similarities and differences in caring motivations by broad demographic groups, but clearly these distinctions are compounded because of the overlap between, for example, being female and also being a wife.

Caring is often long-term and is a changing mixture of challenges and rewards but most of the studies here were cross-sectional making it impossible to be confident about any changes in motivations for caring over time. Importantly this meant we were unable to answer confidently one of our review questions – whether motivations for starting to care for someone with dementia were similar or different to motivations for continuing to care. It may be that carers themselves do not make this distinction in their motivations but future research could perhaps focus specifically on this question to help understand why they continue to care even in adverse situations.

## Conclusions

Future research should include more longitudinal studies and should make within study comparisons between different demographic groups (e.g. ethnic and gender groups) in order give greater confidence in identifying similarities and differences between demographic groups.

Articulating motivations for caring in these studies may also imply some sort of conscious choice by carers but for many caring may not be perceived as a choice but perhaps something that happens gradually in the context of mutual caring and support, especially amongst older spouses. Understanding the development of this, often mutual, caring deserves more research.

It is perhaps tempting to focus on dissimilarities between demographic groups – whether these relate to differences in gender or cultural or ethnic background. However, it is equally important to highlight commonalities amongst these groups. The studies included in this review show not only the diversity in motivations for providing care in diverse cultural settings but also the importance of families and the common threads of compassion and love for care recipients. For the participants here, their relationships and their affective bonds pre-dated their caring role [57], emphasising again the similarities across cultures.

Participants here were all already in the caring role but it is also very important to improve our understanding of why people feel unable take on the caring role. The readiness of others in the family and the availability, costs, quality and suitability of residential care and other formal care are obvious factors and are similar to why carers decide whether to move the care recipient into residential care [58]. Future research should investigate this.

## Data Availability

The data are available in the articles included in the review.

## Abbreviations

ASSIA: Applied Social Sciences Index and Abstracts
CINAHL Plus: Cumulative Index to Nursing and Allied Health Literature
CRD: Centre of Reviews and Dissemination
MeSH: Medical Subject Heading
PRISMA: Preferred Reporting Items for Systematic Reviews and Meta-Analysis
UK: United Kingdom
USA: United States of America
